# CCL17、CCL22及CCR4在新诊断多发性骨髓瘤中的表达及临床意义

**DOI:** 10.3760/cma.j.cn121090-20231230-00349

**Published:** 2024-07

**Authors:** 姿帆 肖, 沙沙 邹, 呈风 易, 月 赵, 柳松 吴, 永怀 冯

**Affiliations:** 1 遵义医科大学附属医院血液内科，遵义 563000 Department of Hematology, Affiliated Hospital of Zunyi Medical University, Zunyi 563000, China; 2 南方医科大学第十附属医院（东莞市人民医院）血液科，东莞 523000 Department of Hematology, The Tenth Affiliated Hospital, Southern-Medical University（Dongguan People's Hospital）, Dongguan 523000, China

**Keywords:** 多发性骨髓瘤, CCL17, CCL22, CCR4, 肿瘤发生, Multiple myeloma, CCL17, CCL22, CCR4, Tumorigenesis

## Abstract

**目的:**

研究CCL17、CCL22及CCR4在新诊断多发性骨髓瘤（NDMM）中的表达情况，分析其与临床特征的相关性，初步探讨其在NDMM发生发展中的作用。

**方法:**

选取2020年7月至2022年12月在遵义医科大学附属医院血液内科就诊的40例NDMM患者以及20名健康志愿者作为研究对象，收集两组外周血、骨髓及骨髓活检组织样本并通过RQ-PCR、ELISA、免疫组化检测NDMM患者的CCL17、CCL22以及CCR4的表达水平。分析NDMM患者骨髓单个核细胞（BMMNC）中CCL17、CCL22及CCR4的mRNA表达水平与患者临床指标的相关性。

**结果:**

NDMM患者BMMNC中CCL17、CCL22及CCR4的mRNA表达水平均高于健康志愿者（对照组）（*P*值均<0.001）；NDMM患者外周血上清和骨髓上清中CCL17、CCL22的蛋白表达水平均高于对照组（*P*值均<0.001）；NDMM患者骨髓活检组织中CCL17、CCL22及CCR4的表达水平均高于对照组（*P*值均<0.05）。CCL17 mRNA在合并贫血、骨质损害、肾损害以及单克隆免疫球蛋白≥30 g/L的NDMM患者中表达水平上升（*P*值均<0.05）；CCL22 mRNA在合并贫血、骨质损害、肾损害的NDMM患者中表达水平上升（*P*值均<0.05）；CCR4 mRNA在合并贫血、肾损害的NDMM患者中表达水平上升（*P*值均<0.05）。

**结论:**

与对照组相比，CCL17、CCL22及CCR4在NDMM患者临床样本中均呈高表达，可能参与了NDMM的发生及发展。

## Abstract (en)

**Objective:**

To study the expressions of C-C class chemokine 17（CCL17）, C-C class chemokine 22（CCL22）, and C-C chemokine receptor 4（CCR4）in newly diagnosed multiple myeloma（NDMM）for analyzing their correlations with clinical features and to preliminarily explore their roles in the development of NDMM.

**Methods:**

The study included 40 patients with NDMM and 20 healthy volunteers from the Department of Hematology of the Affiliated Hospital of Zunyi Medical University from July 2020 to December 2022. Peripheral blood, bone marrow, and bone marrow biopsy tissue samples were collected from the two groups. The expression levels of CCL17, CCL22, and CCR4 in patients with NDMM were analyzed using real-time quantitative reverse transcriptase polymerase chain reaction（RQ-PCR）, enzyme-linked immunosorbent assay（ELISA）, and immunohistochemistry. The mRNA expression levels of CCL17, CCL22, and CCR4 in the bone marrow mononuclear cell（BMMNC）of patients with NDMM were analyzed to assess their correlations with clinical indicators.

**Results:**

The mRNA expression levels of CCL17, CCL22, and CCR4 in BMMNC were higher in patients with NDMM than in controls（all *P*<0.05）. The protein expression levels of CCL17 and CCL22 in peripheral blood supernatants and bone marrow supernatants were higher in patients with NDMM than in controls（all *P*<0.05）. The expression levels of CCL17, CCL22, and CCR4 in bone marrow biopsy tissues were higher in patients with NDMM than in controls（all *P*<0.05）. The mRNA expression level of CCL17 was increased in NDMM patients with combined anemia, bone damage, renal damage, and M protein level ≥30 g/L（all *P*<0.05）. The mRNA expression level of CCL22 was increased in NDMM patients with combined anemia, bone damage, and renal damage（all *P*<0.05）. The mRNA expression level of CCR4 was increased in NDMM patients with combined anemia and renal damage（all *P*<0.05）.

**Conclusion:**

CCL17, CCL22, and CCR4 were highly expressed in clinical samples from patients with NDMM compared to those from controls, and they may be involved in the occurrence and development of NDMM.

多发性骨髓瘤（MM）是以克隆浆细胞在骨髓恶性增殖为特点的疾病，其临床表现为：溶骨破坏、免疫缺陷、血液和尿液中单克隆免疫球蛋白（M蛋白）增高等，其发病率位于血液系统肿瘤的第2位[1]。尽管靶向治疗、免疫治疗和造血干细胞移植等显著改善了MM的疗效，但经过一定的缓解持续时间后，仍不可避免出现复发，目前仍然无法治愈[2]。研究表明，骨髓微环境在恶性浆细胞的分化、迁移、增殖、存活和耐药性中起重要作用[3]。基于肿瘤微环境（TME）治疗肿瘤的新方法对肿瘤进行靶向杀伤，有望为新型抗肿瘤药物的研发奠定基础[4]。因此，研究TME对MM的发生发展具有重要的意义[5]。

近年来，趋化因子成为TME研究的热点，CC趋化因子是10种经典受体的配体，是细胞间通讯的重要组成部分，在TME中的功能至关重要[6]。体内实验已经证明，多种CC趋化因子在肿瘤生长和疾病进展中的作用[7]。CCL17和CCL22是CC趋化因子亚家族成员，CCL17被称为胸腺和活化调节趋化因子（thymus- and activation-regulated chemokine，TARC），CCL22被称为巨噬细胞衍生趋化因子（macrophage-derived chemokine，MDC），CCL17、CCL22与其受体CCR4结合参与肿瘤的生物学特性，对肿瘤细胞的增殖、迁移有一定作用，有望成为肿瘤的诊断标志物及抗肿瘤药物的作用靶点[8]。本研究旨在通过RQ-PCR、ELISA、免疫组化等实验方法检测CCL17、CCL22及CCR4表达水平，并初步探讨其与新诊断MM（NDMM）患者临床指标的相关性。

## 病例与方法

1. 病例：本文收集2020年7月至2022年12月由遵义医科大学附属医院确诊的NDMM患者40例，同时纳入20名健康志愿者（男11名，女9名，年龄18～48岁）作为对照组，收集外周血、骨髓、骨髓活检组织样本及临床资料。患者或家属在入组知情同意书上签字，并通过遵义医科大学附属医院伦理委员会审核（批件号：KLLY-2021-046）。所有患者诊断及分期标准参照《中国多发性骨髓瘤诊治指南（2018年版）》，入选对象排除获得性免疫缺陷综合征、严重器质性疾病及恶性肿瘤患者。

2. 主要仪器与试剂：Varioskan LUX多功能酶标仪、高速冷冻离心机（美国Thermo Fisher Scientific公司），ChemiDoc凝胶成像系统（美国Bio-Rad公司），荧光定量PCR仪（瑞士罗氏公司），PCR引物（上海生工生物工程股份有限公司），CCL17/CCL22酶联免疫试剂盒（上海江莱实业股份有限公司），cDNA合成试剂盒、PCR试剂盒（日本TaKaRa公司），抗CCL17抗体（英国Abcam公司），抗CCL22抗体、抗CCR4抗体（美国Thermo Fisher公司）。

3. 骨髓单个核细胞（BMMNC）提取：无菌条件下采集NDMM组及对照组骨髓样本，300×*g*离心5 min弃去上清，加入等量PBS溶液稀释混匀后缓慢加入到含等量Ficoll淋巴细胞分离液离心管内，400×*g*离心30 min，吸取白雾层离心弃上清形成细胞团块，PBS溶液洗涤2次，−80 °C保存。

4. RQ-PCR：实验选取NDMM组为实验组，健康志愿者为对照组，采用RQ-PCR检测CCL17、CCL22及CCR4的mRNA在BMMNC中表达情况。采用Trizol法提取BMMNC总RNA，使用引物扩增cDNA，使用SYBR Green PCR试剂盒检测CCL17、CCL22及CCR4的mRNA表达。根据CCL17、CCL22、CCR4基因序列由上海生工生物工程有限公司设计及合成引物，使用GAPDH引物作为内参，使用2^−ΔΔCT^方法计算分析各mRNA的相对表达量，引物序列见表1。

**表1:** RQ-PCR引物序列

目的基因	引物序列（从5′到3′）
CCL17-F	GAAGCCTCCTCACCCCAGACTC
CCL17-R	TCTCCCTCACTGTGGCTCTTCTT
CCL22-F	GAAACACTTCTACTGGACCTCA
CCL22-R	TGGCTCAGCTTATTGAGAATCA
CCR4-F	AAATTCTGTGGTGGTTCTGGTCC
CCR4-R	CCGAGATGGCAAGGTTGAGCAG
GAPDH-F	TCAAGAAGGTGGTGAAGCAGG
GAPDH-R	AGCGTCAAAGGTGGAGGAGTG

**注** F：正向；R：反向

5. ELISA：实验步骤严格按照试剂盒说明书进行，外周血及骨髓上清液稀释后作为样品，标准品浓度按说明书的倍比稀释，每孔加入100 µl样品或标准品，37 °C孵育1 h弃去液体，每孔加100 µl生物素化抗体工作液，37 °C孵育1 h，洗板3次，每孔中加入100 µl酶偶联工作液，37 °C孵育30 min，弃液，洗板5次，每孔加入90 µl底物，37 °C孵育15 min，最后每孔加入50 µl终止液，充分混匀。在酶标仪上检测吸光度值，通过计算机软件进行拟合，获得最优拟合曲线。

6. 免疫组织化学染色：切片、烤片、脱蜡、水化，3％的过氧化氢溶液室温避光孵育20 min，加入柠檬酸钠缓冲液，放入高压锅中加热，进行抗原修复，室温自然冷却，滴加血清封闭，加入一抗，4 °C孵育过夜。次日PBS溶液冲洗3次，1次5 min，加入二抗，37 °C孵育15 min，接着滴入适量辣根酶标记链霉素后37 °C孵育15 min，3,3′-二氨基联苯胺（DAB）显色，苏木精复染、脱水、中性树脂封片。结果由2名高年资病理医师采用随机双盲法选择5个高倍视野，结合阳性细胞数目和着色面积来解读，其中阳性细胞的比例评分：<10％计0分、10％～25％计1分、26％～75％计2分、>75％计3分。将阳性细胞比例得分×染色面积得分得到总分，当得分>3分时，定义为高表达，当得分≤3分时，定义为低表达。

7. 统计学处理：采用SPSS 29.0版本进行统计学分析，数据采用*M*（*Q*1，*Q*3）或*x*±*s*表示，两组间均数比较采用*t*检验或非参数检验，多组间比较采用单因素方差分析，采用Fisher确切概率法对多分类数据的多组率进行比较，*P*<0.05为差异有统计学意义。

## 结果

1. NDMM患者临床基线评估：40例NDMM患者，男23例，女17例，中位年龄为62（46～85）岁，≥60岁患者23例。国际分期系统（ISS）分期为Ⅲ期患者33例（82.5％）；Durie-Salmon（DS）分期为Ⅲ期患者33例（82.5％）；贫血32例（80.0％）；合并肾损害患者8例（20.0％）；骨质破坏32例（80.0％）；血小板异常14例（35.0％）；血清校正钙≥2.75 mmol/L 5例（12.5％）；LDH>271 U/L 8例（20.0％）；β_2_微球蛋白≥5.5 mg/L 25例（62.5％）；尿本周蛋白阳性7例（17.5％）；M蛋白≥30 g/L 14例（35.0％）。

2. NDMM组及对照组BMMNC中CCL17、CCL22、CCR4的mRNA表达水平：采用RQ-PCR方法检测表达水平，结果如图1所示，NDMM组BMMNC中CCL17 mRNA相对表达量为7.07±1.27，对照组BMMNC中CCL17 mRNA相对表达量为1.41±0.66；NDMM组BMMNC中CCL22 mRNA相对表达量为23.98±2.65，对照组BMMNC中CCL22 mRNA相对表达量为5.14±1.80；NDMM组BMMNC中CCR4 mRNA相对表达量为6.12±1.81，对照组BMMNC中CCR4 mRNA相对表达量为1.25±0.82。与对照组相比，NDMM组BMMNC中CCL17、CCL22和CCR4的mRNA表达水平均显著增加（*P*值均<0.001）。

**图1:**
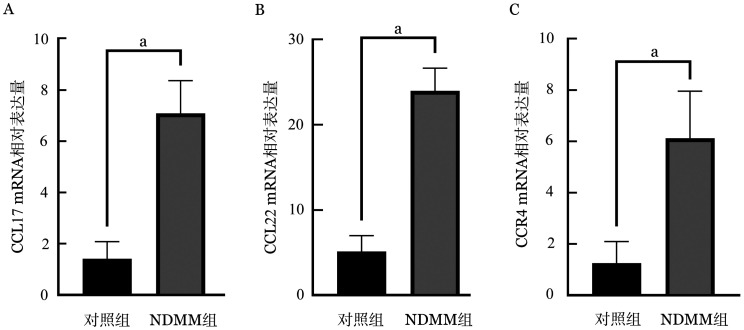
NDMM组与健康志愿者（对照组）BMMNC中CCL17（A）、CCL22（B）、CCR4（C）的mRNA表达水平 **注** NDMM：新诊断多发性骨髓瘤；BMMNC：骨髓单个核细胞；^a^*P*<0.001

3. ELISA法检测NDMM组与对照组中CCL17、CCL22的表达水平：NDMM组外周血上清CCL17中位浓度为1 073.00（833.15，1 569.30）pg/ml，而对照组外周血上清CCL17中位浓度为278.10（229.60，330.90）pg/ml，NDMM组外周血上清CCL17浓度高于对照组（*P*<0.001）；NDMM组外周血上清CCL22中位浓度为1 587.82（1 343.33，3 059.53）pg/ml，而对照组外周血上清CCL22中位浓度为604.95（475.42，724.34）pg/ml，NDMM组外周血上清CCL22中位浓度显著高于对照组（*P*<0.001），如图2所示。NDMM组骨髓上清CCL17中位浓度为1 617.91（1 615.17，2 045.72）pg/ml，而对照组骨髓上清CCL17中位浓度为582.42（349.17，790.83）pg/ml，NDMM组骨髓上清CCL17中位浓度显著高于对照组（*P*<0.001）；NDMM组骨髓上清CCL22中位浓度为12 486.95（8 435.79，17 451.19）pg/ml，而对照组骨髓上清CCL22中位浓度为1 000.22（374.53，1 432.24）pg/ml，NDMM组骨髓上清CCL22浓度高于对照组（*P*<0.001），如图3所示。

**图2:**
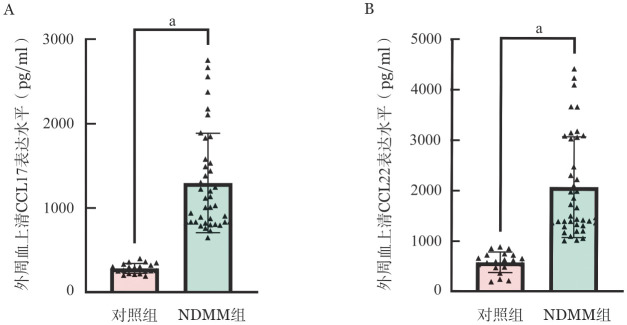
ELISA检测NDMM患者及健康志愿者（对照）外周血上清中CCL17（A）、CCL22（B）的表达水平 **注** NDMM：新诊断多发性骨髓瘤；^a^*P*<0.001

**图3:**
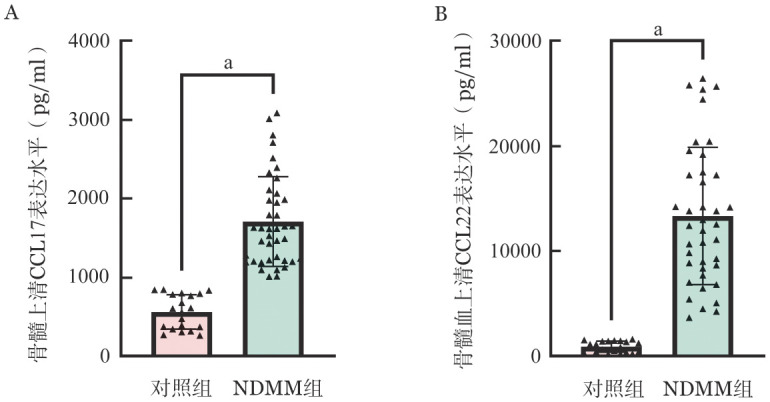
ELISA检测NDMM患者及健康志愿者（对照）骨髓上清中CCL17（A）、CCL22（B）的表达水平 **注** NDMM：新诊断多发性骨髓瘤；^a^*P*<0.001

4. 免疫组织化学染色检测NDMM患者及健康志愿者骨髓活检组织中CCL17、CCL22及CCR4表达水平（图4）：35例NDMM患者中，30例CCL17高表达（85.7％）；而在12名健康志愿者中，CCL17高表达有3例（25.0％）；35例NDMM患者中，32例CCL22高表达（91.4％）；在12名健康志愿者中，CCL22高表达有2例（16.7％）；35例NDMM患者中，30例CCR4高表达（85.7％）；而在12名健康志愿者中，CCR4高表达有2例（16.7％）。NDMM患者骨髓活检组织中CCL17、CCL22及CCR4的表达水平均高于健康志愿者，差异有统计学意义（*P*值均<0.001）。部分患者因未采集到骨髓活检标本，故缺失例数。

**图4:**
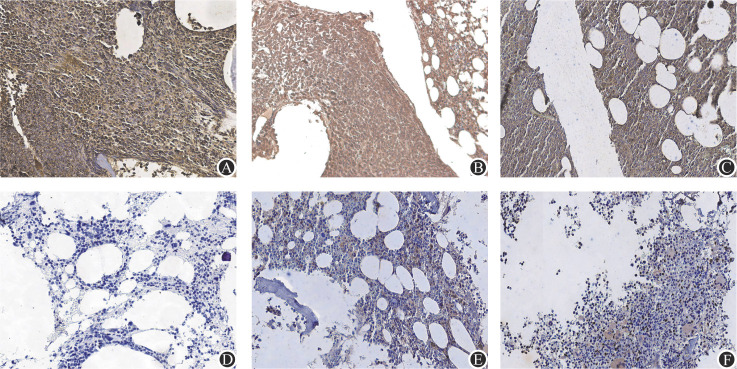
CCL17、CCL22及CCR4在新诊断多发性骨髓瘤（NDMM）患者及健康志愿者骨髓活检组织免疫组化结果（×200） **注** CCL17（A）、CCL22（B）、CCR4（C）在NDMM患者骨髓活检组织中呈高表达；CCL17（D）、CCL22（E）、CCR4（F）在健康志愿者骨髓活检组织中呈低表达

5. NDMM患者BMMNC中CCL17、CCL22、CCR4的mRNA表达水平与临床特征的相关性（表2）：CCL17 mRNA表达水平与贫血、肾损害、骨病、M蛋白具有相关性（*P*值均<0.05），而与性别、年龄、尿本-周蛋白、β_2_微球蛋白、LDH、血清校正钙等无相关性（*P*值均>0.05）。合并贫血、骨病、肾损害的CCL17 mRNA表达水平明显升高（*P*值均<0.05），M蛋白≥30 g/L的NDMM患者BMMNC中CCL17 mRNA表达水平明显升高（*P*<0.05）。

**表2:** 40例新诊断多发性骨髓瘤患者的临床指标与CCL17、CCL22及CCR4 mRNA表达水平的相关性分析

临床资料	CCL17			CCL22			CCR4		
	相对表达量（*x*±*s*）	*t*值	*P*值	相对表达量（*x*±*s*）	*t*值	*P*值	相对表达量（*x*±*s*）	*t*值	*P*值
贫血		4.978	<0.001		2.691	0.015		2.173	0.036
有	7.63±1.09			24.84±2.46			6.54±1.83		
无	5.93±0.72			22.70±2.31			5.25±1.42		
肾损害		4.098	<0.001		3.981	<0.001		2.457	0.019
有	7.71±1.17			25.47±2.15			6.73±1.80		
无	6.30±0.89			22.63±2.25			5.38±1.53		
骨病		5.196	<0.001		2.140	0.039			
有	7.61±1.07			24.65±2.58					
无	5.83±0.66			22.87±2.28					
单克隆免疫球蛋白（g/L）		7.618	<0.001						
≥30	8.06±0.89								
<30	6.09±0.70								

CCL22 mRNA表达水平与贫血、肾损害、骨病相关（*P*值均<0.05），而与性别、年龄、DS分期、M蛋白、尿本-周蛋白、β_2_微球蛋白、LDH、血清校正钙等无相关性（*P*值均>0.05）；合并贫血、肾损害、骨病的NDMM患者CCL22 mRNA表达水平明显升高（*P*值均<0.05）。CCR4 mRNA表达水平与贫血、肾损害相关，合并肾损害的NDMM患者CCR4 mRNA表达水平明显高于无肾损害组（*P*<0.05）。

## 讨论

MM是浆细胞恶性增殖性疾病，其发病机制尚未明确，由此导致的疾病复发、耐药使其无法治愈，是亟待解决的临床难题[9]。近年来，除了靶向肿瘤细胞以外，MM的TME也是研究的热点，TME中肿瘤细胞与微环境中的细胞相互作用介导的肿瘤免疫逃逸被认为参与了MM的发生及进展[3],[10]。越来越多的研究表明，CCL17、CCL22与受体CCR4相互作用促进肿瘤的免疫逃逸，与血液肿瘤的发生、发展和预后密切相关[11]。

本研究结果显示，NDMM患者外周血和骨髓上清中的CCL17和CCL22的表达水平明显高于对照组，NDMM患者BMMNC中CCL17、CCL22及CCR4的mRNA表达水平明显高于对照组。这提示CCL17-CCL22/CCR4轴可能参与了NDMM的发病。MM最为常见的临床表现为“CRAB”症状（高钙血症、肾损害、贫血及骨病），本研究中合并贫血、肾损害及骨病变的患者CCL17和CCL22的mRNA表达水平均高于无以上症状的患者，合并有贫血、肾损害的NDMM患者BMMNC中CCR4的mRNA表达水平明显高于对照组。因此推测CCL17、CCL22及CCR4可能参与了NDMM的发病及进展。NDMM患者的分期、M蛋白、β_2_微球蛋白等指标表提示瘤负荷的高低[12]–[13]。为此本研究也分析了CCL17、CCL22及CCR4的表达水平与这些指标的相关性，结果显示NDMM患者BMMNC中CCL17的mRNA表达水平与高M蛋白水平（≥30 g/L）呈现正相关，这表明CCL17的mRNA表达水平可能还与NDMM肿瘤负荷相关。但是，我们的研究存在不足之处，本研究样本量相对较少，临床样本仅纳入了NDMM患者。

综上所述，研究验证了CCL17、CCL22及其受体CCR4在NDMM患者中的表达水平及其与NDMM患者临床特征之间的相关性，结果表明CCL17、CCL22及CCR4在NDMM患者中高表达。此外，研究还发现在NDMM患者BMMNC中CCL17 CCL22及CCR4的mRNA表达水平与NDMM患者的临床特征相关。因此，本研究推测CCL17、CCL22及CCR4可能参与了NDMM疾病的发生及进展。

## References

[1] Cowan AJ, Green DJ, Kwok M (2022). Diagnosis and management of multiple myeloma: A review[J]. JAMA.

[2] Lei M, Kim EB, Branagan A (2019). Current management and emerging treatment strategies for multiple myeloma[J]. Rinsho Ketsueki.

[3] Kawano Y, Moschetta M, Manier S (2015). Targeting the bone marrow microenvironment in multiple myeloma[J]. Immunol Rev.

[4] Vitale I, Manic G, Coussens LM (2019). Macrophages and metabolism in the tumor microenvironment[J]. Cell Metab.

[5] Ocaña MC, Martínez-Poveda B, Quesada AR (2019). Metabolism within the tumor microenvironment and its implication on cancer progression: An ongoing therapeutic target[J]. Med Res Rev.

[6] Hughes CE, Nibbs R (2018). A guide to chemokines and their receptors[J]. FEBS J.

[7] Korbecki J, Kojder K, Simińska D (2020). CC chemokines in a tumor: A review of pro-cancer and anti-cancer properties of the ligands of receptors CCR1, CCR2, CCR3, and CCR4[J]. Int J Mol Sci.

[8] Ohue Y, Nishikawa H (2019). Regulatory T (Treg) cells in cancer: Can Treg cells be a new therapeutic target?[J]. Cancer Sci.

[9] Shah UA, Mailankody S (2020). Emerging immunotherapies in multiple myeloma[J]. BMJ.

[10] de Jong M, Kellermayer Z, Papazian N (2021). The multiple myeloma microenvironment is defined by an inflammatory stromal cell landscape[J]. Nat Immunol.

[11] Zijtregtop E, Diez C, Zwaan CM (2023). Thymus and activation-regulated chemokine (TARC) as treatment response marker for paediatric Hodgkin lymphoma: A pilot study[J]. Br J Haematol.

[12] Jin Y, Shang Y, Liu H (2019). A retrospective analysis: A novel index predicts survival and risk-stratification for bone destruction in 419 newly diagnosed multiple myelomas[J]. Onco Targets Ther.

[13] Hofste Op Bruinink D, Kuiper R, van Duin M (2022). Identification of high-risk multiple myeloma with a plasma cell leukemia-like transcriptomic profile[J]. J Clin Oncol.

